# Age profiles of sport participants

**DOI:** 10.1186/s13102-016-0031-3

**Published:** 2016-03-12

**Authors:** Rochelle M. Eime, Jack T. Harvey, Melanie J. Charity, Meghan M. Casey, Hans Westerbeek, Warren R. Payne

**Affiliations:** Institute of Sport, Exercise and Active Living, Victoria University, PO Box 14428, Melbourne, Victoria 8001 Australia; School of Health Sciences and Psychology, Federation University, Ballarat, Australia

**Keywords:** Sport, Participation, Age patterns

## Abstract

**Background:**

Participation in sport has many health benefits, and is popular amongst children. However participation decreases with age. While the membership records of peak sports organisations have improved markedly in recent years, there has been little research into sport participation trends across the lifespan. This study investigates age profiles of participation in sport and compares these trends between genders and residential locations.

**Methods:**

De-identified 2011 participant registration data for seven popular Australian sports (Australian Football, Basketball, Cricket, Hockey, Lawn Bowls, Netball and Tennis) were obtained and analysed according to age, gender and geographical location (metropolitan v non-metropolitan) within the state of Victoria, Australia. All data were integrated and sports were analysed collectively to produce broadly based participation profiles while maintaining confidentiality of membership data for individual sports.

**Results:**

The total number of registered participants included in the data set for 2011 was 520,102. Most participants (64.1 %) were aged less than 20 years. Nearly one third (27.6 %) of all participants were aged 10–14 years, followed by the 5–9 year age group (19.9 %). Participation declined rapidly during adolescence. A higher proportion of males than female participants were young children (4–7 years) or young adults 18–29 years; this pattern was reversed among 8–17 year-olds. A higher proportion of metropolitan participants were engaged between the ages of 4–13 and 19–29, whereas a higher proportion of non-metropolitan participants played during adolescence (14–18 years) and throughout mature adulthood (30+ years).

**Conclusions:**

Increasing participation in sport is an objective for both government and sporting organisations. In order to have both mass population-based participation, from a health policy and elite performance perspective, we need to further explore the findings arising from the analysis of this extensive data set. Such an examination will lead to better understand of the reasons for attrition during adolescence to inform program and policy developments to retain people participating in sport, for a healthy and sport performing nation.

## Background

Sport is a common form of Leisure Time Physical Activity (LTPA) [1, 2] which has been shown to result in many health benefits. Recent systematic reviews found that there are many psychological and social health benefits specifically associated with participation in sport for children, adolescents and adults [3, 4]. There is consistent evidence that those who participate in club-based and/or team-based sport participation can have better psychological and social health outcomes than those that only engage in individual types of physical activity (PA) [3, 4]. The social nature of club- and team-based sport is suggested to mediate these health outcomes, although the psychological and social health benefits of sport participation differ between children, adolescents and adults. For children and adolescents social health benefits are more prominent, such as development of social skills through opportunities for social interaction and improved self-esteem, whereas sport participation among adults is more likely to lead to better psychological health, including reduced stress and distress [3, 4]. In addition to the mental and social health benefits, club sport has been shown to be associated with greater physical health benefits at low and moderate levels of participation, than participation in individual-based physical activities such as walking [5]. From a public health perspective, sport during adolescence is a strong predictor of PA later in life [6, 7].

Understanding participation patterns in sport is also important for a range of key stakeholders including government, sport and recreation, and health organisations, and in particular sport governing bodies [2]. Population-level sport participation patterns can inform evidence-based strategic and policy planning and development [8, 9] and facilitate the achievement of desirable outcomes. For instance, in Australia the National Sport Policy Framework provides a guide outlining the importance of sport policies and coordinated strategies at both the community and elite levels for increased participation and a healthy nation, as well as for international success of elite athletes [10].

Sport participation patterns are typically explored according to age and gender. There is evidence that sport participation is a young persons’ activity [11], with reports that participation levels peak at ages 12–13 years [12]. However, others have found that for Belgian boys aged 13–18 years, there was a linear increase in sport participation until age 16.8 years, before participation declined [13]. Another study of sport and PA participation by girls found that overall PA levels did not significantly change throughout adolescence, but that the context of participation changed [14]. Older adolescent females (16–18 years) shifted their participation away from organised, competitive modes and settings towards non-organised and non-competitive modes and settings and were more likely to then participate in individual types of PA [14].

For adults, the relationship between sport participation, age and gender has been found to differ amongst European countries [15]. In France, Latvia, Slovakia and the UK, males reported significantly more sport participation than women in the young adult age group (18–34 years). In Belgium and Greece, males were more likely to participate than females in both the young adult (18–34 years) and older adult (55 years and older) categories [15]. In contrast, Swedish women were more active than males in the young adult category (18–34 years); whilst in Finland this was only true for the middle-age group (35–54 years) and in Denmark for the older adults (55 years and older) [15]. In Australia, sport participation in an organised context was dominated by those aged 15–34 years compared to all older age groups, for both males and females [2]. Similarly in Spain, the prevalence of participation in sport decreased as age increased [16]. Amongst older adults aged 58–67, sport participation has also been found to decrease with age [17].

There are significant gender differences in sports participation in European countries, where males were more likely to participate in sport more regularly than females in Belgium, France, Greece, Latvia, Lithuania, Slovakia, Spain and the UK, whilst the opposite was true for Denmark, Finland, Sweden and the Netherlands [15]. The authors point out that historically male participation in sport has dominated over female participation, however some policy developments targeted at increasing participation in sport for females may have contributed to higher participation rates for females in some countries [15]. For instance in Belgium, available data show a greater level of male than female participation, females have closed the gap considerably since the 1970s [18]. However, with regard to the club based, organised context of sport participation, there were no gender differences detected in the Belgian study [18]. Amongst older Dutch adults, males and females were equally likely to participate in sport, or to be a sports club member, however participation in competition was more likely to occur amongst males rather than females [17].

In general, the above research provides evidence that as age increases participation in sport decreases. However, these studies are often limited to self-report sample surveys and/or to specific age ranges. Furthermore, most studies do not compare different residential locations. It is important to better understand participation in sport and how it relates to age, gender and geographical location, in order to inform evidence-based, well targeted program and policy development. The aim of this study was to use a unique, very large set of comprehensive membership registration data, effectively a census of participation, to provide age profiles of participation across seven major sports [14, 19], across the lifespan, and to compare these trends between genders and residential locations.

## Methods

We investigated profiles of sport participation according to age, gender and geographical region in the Australian state of Victoria. As part of the Sport and Recreation Spatial project (www.sportandrecreationspatial.com.au) the authors of this study have initiated a large repository of sport registration data to inform evidence-based decision making across the sport sector. The seven sports incorporated in the study (Australian Football, Basketball, Cricket, Hockey, Lawn Bowls, Netball and Tennis) include six of the 10 most popular adult club-based physical activities in Australia, and five of the 10 most popular organised physical activities for children [20]. De-identified data on participant registrations were obtained from the respective state sporting associations (SSAs), the sports’ state governing bodies. In this way the study was able to overcome the limitations of many studies in this area, such as the use of self-reported data, narrowly defined player population segments and failure to examine geographical variation.

Of the seven SSAs engaged in the study, five register participants for a calendar year with age determined at 1 January, and two register participants for a financial year, with age being calculated as at 1 July. The scope of this study was nominally the calendar year 2011; we included 2011 registrations for five sports and 2011–12 registrations for two sports. Ethics approval was granted by the Federation University Australia Human Research Ethics Committee.

All data were integrated and sports were analysed collectively in order to produce broadly based participation profiles while maintaining confidentiality of membership data for individual sports. An individual could engage in more than one sport and was counted separately in each sport, with the result that counts of participants are to some extent weighted by individuals’ levels of participation. Because of anonymity provisions, it was not possible to identify participation of a particular individual in more than one sport, but based on an analysis of demographic characteristics and residential postcodes, the proportion of individuals who were registered in more than one sport was estimated to be around 12 % across Victoria. The methods of data integration and analysis have been reported previously [21].

Regional breakdowns were based on residential postcodes. Although postcode areas are not precisely geographically specified, the Australian Bureau of Statistics (ABS) precisely defines approximations to postcode areas entitled postal areas [22]. ABS also produces a correspondence table for assigning population-weighted proportions of postal areas to local government areas (LGAs) [23], which enabled estimated numbers of participant registrations in each LGA to be calculated. The 79 Victorian LGAs are classified as metropolitan (31 LGAs) or non-metropolitan (48 LGAs) by the Victorian state government [24].

It is important to emphasise that the age profiles reported throughout this paper are based on proportions of all participants, not on age-specific participation rates. The outcome variable, the proportion of participants in an age category, was defined as the number of registered members in that age category, expressed as a percentage of the total number of participants of all ages. This resulted in standardised profiles that could be directly compared between genders and regions. The alternative approach of expressing each number of participants as a proportion of the population in the same age category (i.e. a participation rate) would result in profiles with different scales due to different overall participation rates for the different genders and regions. Furthermore, ABS inter-censal population estimates are only published for 5-year age cohorts, whereas there is no such limitation for participant age profiles because the birthdate, and hence the exact age, of each participant is known.

Two sets of age profiles were tabulated and graphed. The first covers the whole life span, tabulated in 5-year age ranges and graphed in single-year age ranges, and the second provides a more detailed single-year picture of the 4–29 year age range. In each case, overall age profiles and separate profiles for each gender and region are presented.

Because data were collected from the whole population of members of each sport, i.e. a census rather than a sample, statistical inference was not applicable. Analyses were conducted using Excel and SPSS Version 21.

## Results

The seven sports have been de-identified in all results presented. While a small proportion of participants registered in Victoria resided in other states, only those residing in Victoria were included. After consultation with SSAs, the valid age range was considered to be 4–100 years. Those for whom no birthdate were recorded (*n* = 34,341; 6.2 %) were excluded from the analysis, as were those whose calculated ages at the appropriate registration date were outside the valid age range (*n* = 974; 0.2 %). The remaining number of registered participants in the seven sports ranged from 13,275 to 171,304, with the total number of registered participants for 2011 being 520,102.

### Overall trends

Table 1 provides age profiles of registered sport participants, and breakdowns by gender and region, within standard ABS 5-year age cohorts (plus a separate 4-year-old cohort). Most participants (64 %) were aged less than 20 years. Nearly one third of all participants were aged between 10–14 years (27.6 %), followed by the 5–9 year age group (19.9 %) and the 15–19 year age group (15.3 %). Fewer than 10 % of participants were over the age of 50 years. Given that the great majority of participants (79.1 %) in the seven sports were aged from 4–29 years more detailed age profiles are presented in Table 2.

**Table 1:** Age profiles of registered sport participants: percentage of total sport participants

Age (years)	Participants (%)	Male (%)	Female (%)	Metropolitan (%)	Non-Metropolitan (%)
4	1.3	0.5	1.7	1.5	0.9
5–9	19.9	17.1	21.5	21.5	17.0
10–14	27.6	32.1	25.6	28.1	26.7
15–19	15.3	15.8	15.1	14.7	16.5
20–24	8.8	7.8	9.1	9.0	8.5
25–29	6.1	5.4	6.3	6.3	5.8
30–34	4.1	3.8	4.2	4.0	4.2
35–39	3.0	3.5	2.8	2.9	3.4
40–49	5.0	6.0	4.6	4.9	5.2
50–59	2.5	2.5	2.6	2.3	3.0
60–69	2.6	2.6	2.6	2.0	3.8
70+	3.6	3.0	3.9	2.8	5.1

**Table 2:** Age profiles of registered sport participants aged 4–29 years: percentage of total sport participants

Age (years)	Participants (%)	Male (%)	Female (%)	Metropolitan (%)	Non-Metropolitan (%)
4	1.3	1.7	0.5	1.5	0.9
5	3.0	3.8	1.4	3.3	2.5
6	3.5	4.1	2.2	3.8	2.8
7	3.8	4.2	3.2	4.2	3.2
8	4.5	4.5	4.6	4.8	4.0
9	5.0	4.8	5.6	5.3	4.5
10	5.9	5.4	6.9	6.2	5.4
11	5.9	5.4	6.9	6.1	5.5
12	5.7	5.3	6.4	5.7	5.6
13	5.3	4.9	6.2	5.3	5.3
14	4.9	4.6	5.6	4.8	5.0
15	4.2	3.9	4.8	4.0	4.5
16	3.6	3.4	4.0	3.3	4.0
17	2.8	2.8	2.9	2.6	3.2
18	2.5	2.6	2.2	2.4	2.5
19	2.3	2.4	2.0	2.3	2.2
20	2.1	2.1	1.9	2.1	2.0
21	1.9	2.0	1.7	1.9	1.9
22	1.7	1.8	1.5	1.8	1.7
23	1.6	1.7	1.4	1.7	1.5
24	1.5	1.5	1.3	1.5	1.4
25	1.4	1.5	1.3	1.5	1.3
26	1.3	1.3	1.2	1.3	1.2
27	1.2	1.2	1.1	1.2	1.1
28	1.1	1.2	1.0	1.2	1.1
29	1.0	1.1	0.9	1.1	1.0
Total % in 4–29 year age range	79.1	79.3	78.6	81.2	75.4

### Gender

**Fig. 1:**
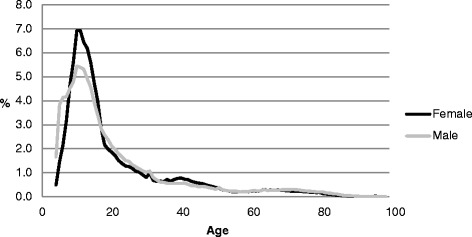
Age profiles by gender

**Fig. 2:**
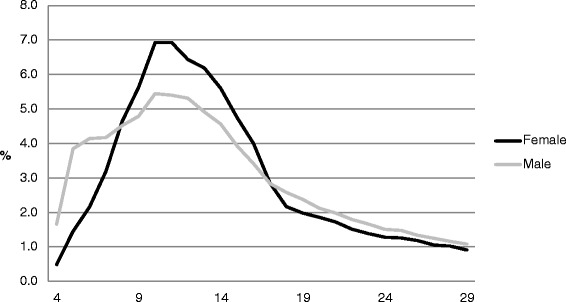
Age profiles (4–29 years) by gender

Tables 1 and 2 and Figs. 1 and 2 show that, in terms of sport participation by gender, a higher proportion of male participants were very young (4–7 years) (13.8 %) compared to females (7.3 %); this pattern was repeated in young adulthood (18–29 years; 20.4 % and 17.5 % for males and females, respectively). A higher proportion of female participants were aged 8–17 (53.9 %) than males (45.0 %). The proportion of female participants aged 30–49 was again slightly higher compared to males. Beyond age 50, gender differences were negligible.

A deeper examination of the data revealed that the highest proportion of participants for both males and females was in the 10–11 age group (5.4 % and 6.9 % for males and females, respectively). For all age groups with a proportion of participants of 4 % or more, the males were younger (aged 6–14) than the females (aged 8–16). This represented a 9-year span for both genders. A much higher proportion of male participants were aged 4–5 (5.5 %) than was the case for females (1.9 %).

### Region

**Fig. 3:**
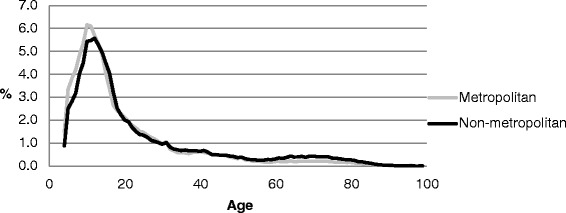
Age profiles by geographical region

**Fig. 4:**
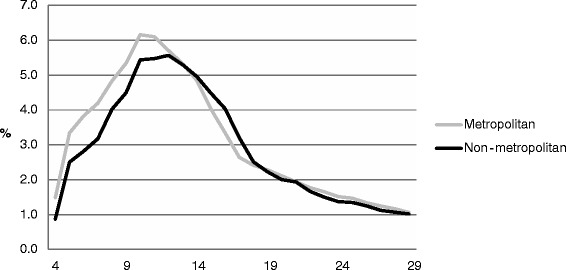
Age profiles (4–29 years) by geographical region

Tables 1 and 2 and Figs. 3 and 4 provide details of the age profiles by geographical region. Higher proportions of metropolitan than non-metropolitan registered sport participants were engaged in the seven sports between the ages of 4–12 and ages 19–29; whereas higher proportions of non-metropolitan registered participants were engaged during adolescence (14 – 18 years) and throughout most of adulthood (30+ years).

A closer analysis of the 4–29 year age group, where around 80 % of the participation occurs, showed some key differences in age between metropolitan and non-metropolitan registered sport participants. The ages of 4–12 years represented a higher proportion of all metropolitan registered sport participants than for non-metropolitan. This difference was reversed from ages 14–18 years onward.

## Discussion

This study is unique in providing population-derived age profiles of club sport participation within popular organised sports in Australia. A strength of this study is in the number of total participants (*n* = 520,102 member records), representing the whole population of registered participants for seven popular sports in the state of Victoria, Australia in 2011. However, it must be noted that this does not include all participants in these sports, such as those participating in school based programs or ‘involved’ participants for example those engaged in a non-physical manner such as coaches and officials [11].

Participation in club sport is clearly an activity dominated by young people. The peak representation for both males and females was 10–11 years. However, the 9-year age range across childhood for which the representation for each year of age was greater than 4 % was younger for males (6–14 years) than for females (8–16 years). Furthermore, there were considerably more males participating aged 4–5 years (6 %) than females at these ages (2 %).

Very few studies have provided age profiles of all registered participants in a sport; rather, the proportion of a study sample in sport at a given time has been reported [16, 25]. Nevertheless, other studies in Australia have reported that sports participation peaked at 12–13 years for boys and girls and fell by 50 % by age 16 [12]. In a national Australian population survey, it was reported that in the 12 months prior to the survey 60 % of all children aged 5 to 14-years participated in at least one organised sport activity outside of school hours [19]. The participation rates were 56 % for 5–8 year olds, 66 % for 9–11 and 60 % for 12–14 year olds [19]. While the present study focuses on the age profile rather than participation rates, our results are consistent with the 2012 study; both report a lower peak participation age than the 2009 study. This may be the result of a growing trend to get children engaged in organised sport at a younger age via the increasing availability of modified sports programs. However the apparent increase in younger participants in sport and especially rising numbers in 4–5 year olds raises another issue. Younger children tend to sample sports, that is they typically play several different sports, and only when older, around 13 years, do they tend to specialise and focus their attention on one sport [26]. This is also the age when they are most likely either become involved in organised competitive sport or drop out of sport altogether [27]. However, the apparent tendency towards earlier participation in modified programs, and the consequent longer period between commencement and preparedness for specialisation and competition, may lead to a greater risk of dropout, either through boredom with an extended period in modified programs, or because of the temptation and/or pressure to transition prematurely into standard competitive forms of sport before their capacities are adequately developed [21]. Is there a risk that the specific targeting of very young children could have a negative effect on long term sport participation and potentially on social, mental and physical health outcomes? Further research is required to investigate whether the participation of very young children leads to higher or lower uptake of (organised) competitive sport later in childhood, and any affect that this may have on their health.

The reason that a greater proportion of males participate at a younger age than females is not known, however it may relate to boys being encouraged more to participate in sport at a young age than girls. There is some evidence to suggest that in families with male and female children, fathers tend to be more involved with their sons’ sport than their daughters’ [28]. Furthermore, parents influence children’s sport participation [29] and this is shaped by the parents’ own sporting backgrounds [28]. Since adult males are more likely to participate in sport than females [2], this may be a factor in encouraging the participation of young boys more than young girls.

While the development of modified sports programs for young children provide sporting opportunities to a new, wider, consumer segment, there are fewer opportunities for adolescents or adults to participate in sport outside standard club competition. Generally, sporting opportunities for children in the 10–14 age group in Australia and other nations such as the UK and Germany are focused on standard club competitions. Our study showed that the participation peaked for both males and females at age 11, and nearly a third of participants were aged between 10–14 years. This raises the question as to whether the available sport programs/opportunities are meeting the needs of adolescents and adults. Research has shown that whilst for those aged 15 years and older participation in leisure-time physical activity significantly increased over a 10 year period, participation in organised and/or club sport remained relatively stable [2]. It is evident that there are many individuals who wish to be active in their leisure-time, and health is often a major driver, but their interest in being physically active shifts away from traditional (organised, competitive) sport [2, 30]. Participation in organised leisure-time activities (such as sport) is not as popular as non-organised activities among those aged 15 years and over, with roughly 27 % participating in organised activities compared to 53 % participating in non-organised activities at least once within a 12-month period [19]. It seems that the drop-off in sport participation during adolescence towards non-organised activities occurs at the same time when people would enter the elite sport pathway. Indeed, it has been suggested that as young people progress through early- to mid-adolescence, there are fewer and fewer opportunities to play sport for all but the most able and mature [31].

From an elite sport policy perspective, fewer individuals participating after the age of around 12 years would make the pool available for talent identification and elite pathway progression smaller. Researchers suggest that “talent identification models are likely to exclude many, especially late maturing, ‘promising’ children from development programmes due to the dynamic and multidimensional nature of sport talent” [32]. From a health policy perspective, fewer people active through sport may contribute to a range of negative health outcomes [33].

Fewer than ten per cent of the registered sport participants were adults aged 50 years and over. Exercise, fitness and team sports make up a greater proportion of the moderate and vigorous physical activity of young adults (16–24 years) compared to older adults (65+) [34]. A recent study found that, across both genders, there was no significant difference in the proportion of club sport participants across the ages from 15 to 75+ [2]. However for younger females aged 15–34 participation was significantly higher than all other age groups (35–54, 55–74, and 75+ age groups), which did not differ significantly from each other [2]. As an ageing society, we should consider strategies to increase participation in sport for adults and older adults. In doing so, modifications may be required in sport products and equipment. There are many health benefits of sport participation for older adults, and many participate for social reasons, and enjoy playing with family members across generations [35].

In addition to age-related differences there were some notable geographic differences. A higher proportion of metropolitan children aged 4–13 participated in sport compared to non-metropolitan. The peak metropolitan participation age was younger, at age 10–11 years, compared to non-metropolitan, which peaked at 11–12 years. This may be related to the earlier provision (and a broader range of choice) of modified sports programs in metropolitan regions. However, adults and older adults represented a higher proportion of non-metropolitan than metropolitan participants. This may relate to the central role of community sport in Australian regional communities, where sport can be considered the ‘social cement’ [36], contributing to local identity [37], being ‘held up as gauges of the health of country communities’ [38], and crucial for increasing social connections and community cohesion [39]. It has been reported that in rural and regional areas, participation in sport for adolescent girls is a socially and culturally privileged activity, whereby involvement or lack of participation in club sport positions girls on one side of the physically active/inactive binary categorisation [40]. This is sometimes related to perceived level of competency and lack of choices of activities in rural and regional areas.

Sports governing organisations generally have a strategic goal of increasing the level of participation within their sport. However, operationally their focus is often on development of the player/athlete segment of the population rather than recreational participants. Similarly, sport policy and funding in Australia, and other countries such as the UK and Canada, have been entrenched and focused on the elite level, often prioritising high performance over mass participation [41]. For example, an international analysis of government sport policies revealed that despite the existence of ‘sport for all’ policies emerging in a number of countries, these policies do not enjoy significant infrastructure or human resources support from governments to operationalise the policies, and in particular, do not enjoy the existence and stability of national institutes for elite sport [41]. The development of recreational pathways for sport participants therefore, requires a system-wide approach with cross-sectoral engagement of government and the sport and recreation industry if policy and organisational goals such as increasing sport participation are to be achieved. Ireland is one country that has proposed a hybrid model for participant/player development which not only maps out player/athlete development but also maps recreational pathways to ensure lifelong involvement in sport and physical activity [42]. Finland and the Netherlands also have a political and funding bias towards mass participation, and as a consequence have reported high participation rates compared to Australia, England, Canada and a number of European countries [41]. However, the definition and measurement of what constitutes sport participation varies between nations, which makes it difficult to make comparisons [41].

Redirecting sport policy and funding towards mass participation and the development of recreational pathways is one strategy to increase population levels of sport participation, and promote the health of individuals and communities. In this approach sporting organisations would need to be supported by government to monitor sport participation levels and the impact of sports programs. In another approach, the interests and needs of various population groups require consideration by governments and sporting organisations. However, it is much easier to argue changes to pathways and policies based on theory than it is to determine what are the critical process levers, community interests and stakeholders that will drive those changes. Researchers have suggested that socio-ecological models taken from physical activity research, and sport development concepts taken from sport management theory, be integrated to enhance efforts to increase sport participation [8, 9]. This is particularly important as the determinants of sport participation are often examined within the broader context of leisure-time physical activity [43]. Most studies have examined sport participation focusing on adolescents [44–46]. For instance, a Canadian study of adolescent sport participation identified the need to fit sport into the “time-challenged, gender-stereotyped, highly technologized, cyber-filled lives of today’s youth” [44]. Others have found that different sports have diverse participation determinants, particularly in regard to demographic and economic variables [47]. For instance, in a German study, swimming participation was positively influenced by being young or old (U-form relationship), female, well-educated and a native of the country; whilst football (soccer) participation was positively influenced by being young, male, less-educated and having a foreign nationality [47]. Individual and unorganised physical activities such as running, fitness/gymnasium and going for a walk or hiking were associated with middle age [47]. In Australia, market segmentation studies were recently conducted to better understand the Australian community’s participation in sport and physical activities for both adults (aged 14–65) and children (aged 5–13 years) [48]. Consumer segments amongst the population were characterised to identify challenges and opportunities to engage and/or re-engage individuals in sport. For example, among children segments considered as potential sport participants included ‘thrifty enthusiasts’ who are positive about physical activity, but may not participate in activities organised in a community club; and ‘ponderers’ who would like to do more sport but are unsure how to get involved [48]. The underlying motivation for many sports participants, and especially young people is to have fun and socialise [39, 49]. Key factors that affect participation in sport include: 1) sport delivery that focuses on competition rather than fun and enjoyment; 2) a lack of flexibility around traditional sport club scheduling; 3) teams organised on the basis of talent rather than friendship groups; 4) limited opportunities for those with less sports competency; and 5) self-consciousness amongst adolescents, embarrassed by their lack of sporting ability [48].

This research on the determinants of sport participation and market segmentation raises some important questions regarding the delivery of sport and recreation. For instance, we contemplate whether organised sport can be modified in ways to cater for the needs of adolescent and adults, such as smaller fields, reduced time commitment, or flexibility in player rotations. In doing so, appropriate marketing would be required, as this has been a strength of modified sports programs for children [11]. The voluntary nature of the delivery of sport also needs to be considered in planning and implementing such programs. Others have suggested that alliances with more powerful organisations, particularly in the area of health, are required before sufficient resources can be allocated to promote mass participation [41] and that further collaboration between public health and sport management is required [2, 8].

## Conclusion

In conclusion, this study is the first to examine the age profile of all registered participants in a major sub-national geographical region within seven popular organised sports, across the lifespan. The majority of organised sport participation within these sports was by children aged 10–14 years, peaking at ages 10–11. The proportion of individuals engaged in these sports declined rapidly during adolescence, which may have health implications. Furthermore males and metropolitan participants were more likely to be represented in younger age categories compared to non-metropolitan participants.

Governments and sporting organisations alike have strategic and policy objectives to increase sport participation/mass participation; although these objectives are not sufficiently supported through the provision of infrastructure or resources that measure and analyse participation. A twin track approach to mass participation and elite performance may be required to achieve increases in sport participation as suggested by others [50], such as exemplified in Ireland [42]. In order to implement a twin track approach, further research is required to investigate reasons for attrition more closely especially relating to the very young participant cohorts, to inform program design and to test the efficacy of such programs for promoting an active and healthy nation.

## Abbreviations

ABS: Australian Bureau of Statistics
LGA: local government areas
LTPA: leisure time physical activity
PA: physical activity
SSA: state sporting associations
